# Non-Invasive Multi-Gas Detection Enabled by Cu-CuO/PEDOT Microneedle Sensor

**DOI:** 10.3390/s24113623

**Published:** 2024-06-04

**Authors:** Arif Ullah Khan, Muhammad Tahir, Fazal Ul Nisa, Mizna Naseem, Iqra Shahbaz, Zeyu Ma, Zilu Hu, Abdul Jabbar Khan, Muhammad Sabir, Liang He

**Affiliations:** 1 State Key Laboratory of Intelligent Construction and Healthy Operation and Maintenance of Deep Underground Engineering, School of Mechanical Engineering, Sichuan University, Chengdu 610065, China; au46884@stu.scu.edu.cn (A.U.K.); fazalulnisa@outlook.com (F.U.N.); miznanaseem@gmail.com (M.N.); mazeyu@stu.scu.edu.cn (Z.M.); huzilu@stu.scu.edu.cn (Z.H.); 2 School of Mechatronical Engineering, Beijing Institute of Technology, Beijing 100081, China; 3 Key Laboratory of Green Printing, CAS Research/Education Centre for Excellence in Molecular Sciences, Institute of Chemistry Chinese Academy of Sciences (ICCAS), Beijing 100190, China; iqrashahbaz@iccas.ac.cn; 4 College of Chemistry and Chemical Engineering, Huanggang Normal University, Huanggang 438000, China; 5 State Key Laboratory of Advanced Technology for Materials Synthesis and Processing, Wuhan University of Technology, Wuhan 430070, China; sabirkhan07009@gmail.com; 6 Med+X Center for Manufacturing, West China Hospital, Sichuan University, Chengdu 610041, China; 7 Yibin Industrial Technology Research Institute, Yibin R&D Park, Sichuan University, Yibin 644005, China

**Keywords:** vapor-phase polymerization, non-invasive sensing, environmental monitoring, PEDOT, volatile organic compound

## Abstract

Metal-oxide-based gas sensors are extensively utilized across various domains due to their cost-effectiveness, facile fabrication, and compatibility with microelectronic technologies. The copper (Cu)-based multifunctional polymer-enhanced sensor (CuMPES) represents a notably tailored design for non-invasive environmental monitoring, particularly for detecting diverse gases with a low concentration. In this investigation, the Cu-CuO/PEDOT nanocomposite was synthesized via a straightforward chemical oxidation and vapor-phase polymerization. Comprehensive characterizations employing X-ray photoelectron spectroscopy (XPS), scanning electron microscopy (SEM), X-ray diffraction (XRD), and micro Raman elucidated the composition, morphology, and crystal structure of this nanocomposite. Gas-sensing assessments of this CuMPES based on Cu-CuO/PEDOT revealed that the response current of the microneedle-type CuMPES surpassed that of the pure Cu microsensor by nearly threefold. The electrical conductivity and surface reactivity are enhanced by poly (3,4-ethylenedioxythiophene) (PEDOT) polymerized on the CuO-coated surface, resulting in an enhanced sensor performance with an ultra-fast response/recovery of 0.3/0.5 s.

## 1. Introduction

The continuous evolution of microsensors has ushered in the era of unprecedented innovation, enabling the detection and quantification of various pollutants with remarkable precision and sensitivity in environmental monitoring. Among the diverse sensing platforms, microsensors have emerged as valuable tools for the real-time monitoring of volatile organic compounds (VOCs), which play a significant role in solutions for environmental pollution and human health risks. Acetone is a common chemical compound in the medical realm. However, if its concentration reaches a certain value, it will cause several negative effects on human beings, such as respiratory stimulation, vomiting, and spasms [1,2]. Acetone can also be used as a biomarker for diagnosing diabetes [3]. The principle is that the acetone’s concentration in the exhaled breath of healthy individuals is lower than those with diabetes [4]. People with diabetes exhale about 1.8–10 ppm of acetone gas, whereas people without diabetes exhale less than 0.8 ppm [5]. Therefore, the highly selective and fast detection of acetone below the critical ppm level is crucial for its industrial applications and the early diagnosis of diabetes. Gas-sensing technology is effective in real-time gas detection, thus, this technology is often used to monitor air quality, food freshness, and human health [6]. To meet practical application requirements, gas sensors should have an excellent sensing performance, such as selectivity, sensitivity, and a rapid response, which are closely related to the sensing materials. Among them, semiconductors are the most common due to their low cost, large-scale processability, and good compatibility with microelectronic technology [7,8]. Therefore, many metal-oxide-based semiconductors have been applied as gas-sensing materials for VOCs [9,10,11,12].

The VOCs are a diverse group of chemical compounds that readily vaporize into the atmosphere, originating from both natural and anthropogenic sources. Common sources of VOC emissions include industrial processes, automotive exhaust, agricultural activities, and indoor pollutants such as cleaning agents and building materials. Despite their ubiquitous presence, VOCs pose significant environmental and health hazards, contributing to air pollution, smog formation, and adverse respiratory effects in human beings. Hence, there is an urgent need to detect these hazardous gases at low concentration to realize a safe and healthy environment for living beings [13]. Most of the methanol production is generated from natural gas, which links directly to the greenhouse gas effect, its increasing concentration in the atmosphere poses serious effects, and developing its accurate sensing methods is highly essential [14,15,16,17,18,19,20]. Hence, they must be detected at a low concentration to minimize the adverse effects on human health. Moreover, it becomes dangerously explosive when the concentration of VOCs in the environment reaches about 5–15% [21,22]. Moreover, other important consequences of methane (CH_4_) gas arise from natural gas leakage through pipelines, leading to severe consequences if not identified and detected at a low concentration [23,24].

The accurate detection and monitoring of VOCs are essential for assessing air quality, identifying pollution sources, and implementing effective mitigation strategies [25,26]. Metal nanoparticles, including Au, Ag, Al, and Cu, are believed to be the excellent candidates for integrated devices [27]. Conventional analytical techniques, such as gas chromatography-mass spectrometry (GC-MS) and infrared spectroscopy (IS) offer high sensitivity and selectivity, but they are often limited by their complexity, cost, and inability to provide real-time measurements [28]. As such, there is a growing demand for portable, cost-effective, and user-friendly microsensor platforms capable of the in-situ detecting of VOCs, particularly in urban and industrial settings where exposure levels may exceed regulatory limits. In recent years, metal-oxide-based materials have been extensively used as chemiresistive microsensors for monitoring environmental methane, and the sensing behaviors are the outcomes according to the changes in their electrical/capacitive or optical performance [29,30,31].

In this research, we explored a novel microsensor architecture, like Cu-CuO/PEDOT-based microsensor, combining copper (Cu), copper oxide (CuO), and conductive polymers for improved sensing performance. The vapor phase polymerization with poly (3,4-ethylenedioxythiophene) (PEDOT) obtains an enhanced electrical conductivity and interaction with analytes, boosting sensitivity and selectivity. The factors affecting the microsensor’s performance include the target gas, operating conditions, and architecture. CuO-based microsensor has advantages in sensing reducing gases like methanol, ethanol, ammonia, and acetone. The detection is essential for environmental and industrial safety. Cu-CuO/PEDOT microsensor offers enhanced electrical conductivity and selectivity, particularly to VOCs, with fast response. Their layered structure allows a tailored sensing performance. Cu-based microsensor demonstrates competitive performance in sensitivity, selectivity, and stability. A multifunctional Cu-based multifunctional polymer-enhanced sensor (CuMPES), that is, a Cu-CuO/PEDOT microsensor, shows high promise for VOC detection. The proposed CuMPES was fabricated via chemical oxidation and vapor-phase polymerization on copper wire’s edges, which incorporated a gel polymer electrolyte for the improved interaction with VOCs. The CuMPES exhibited rapid response/recovery of 0.3/0.5 s and high selectivity, indicating its great potential in diverse gases’ environmental monitoring.

## 2. Materials and Methods

The SH Electric Co., Ltd. (Shanghai, China) was the source of the pure copper mesh. The supplier of the lithium chloride (LiCl) was SC. Reagent Co. We purchased polyvinyl alcohol (PVA) from Aladdin Crystallin BT Co., Ltd. We bought sulfuric acid (H_2_SO_4_) and methyl ethyl ketone (MEK) from Aladdin. Every chemical was purchased and utilized without any additional purification. The analytical-grade substances of acetonitrile, acetone, methanol, ethanol, lithium chloride (LiCl), and magnesium chloride (MgCl_2_) were employed without additional purification. Using Milli-Q water from a Millipore system (18 mΩ·cm), aqueous solutions were made.

### 2.1. Synthesis of CuMPES Electrodes

To get rid of organic materials, copper wires with a diameter of about 70 µm were cleaned using isopropyl alcohol (IPA) and deionized (DI) water. We prepared an acceptable chemical stripping solution by diluting a reasonable amount of methyl ethyl ketone (MEK) solvent with DI water in a well-ventilated room to remove polymer cladding from the margins of the copper wire’s surface. To ensure that the edges of copper wire were completely covered, the edges were then submerged in the prepared stripping solution. After the wire was soaked in the solution for 12 h, it was checked regularly. The stripping process was improved by gently agitating the copper wire in the solution once every hour. We took the wire out of the solution after the polymer had sufficiently dissolved and softened, then we thoroughly rinsed it with DI water to get rid of any last bits of residue. Before being used again, the copper wire was finally dried with a fresh cloth and given time to dry in the air entirely.

### 2.2. Preparation of Cu-CuO Electrode

The edges of Cu wire were partially oxidized using the previously reported method [32]. To make an 8 M NaOH solution, 3.2 g of NaOH solid in a beaker was mixed with 10 mL of DI water, followed by the agitation of 10 min until the mixture became clear. Then, the mixture was gradually cooled down to room temperature. An electronic balance was used to weigh 0.913 g of (NH_4_)_2_S_2_O_8_ powder in a different beaker with the appropriate volume. A 0.2 M (NH_4_)_2_S_2_O_8_ solution was obtained when 20 mL of DI water was added and agitated for 10 min, or until no visible undissolved particles were left. The cooled NaOH solution was then gradually added, drop by drop, while stirring to guarantee homogeneity into the (NH_4_)_2_S_2_O_8_ solution, producing the alkaline oxidative etchant solution (AOES) for copper wire etching. After cleaning the copper wires from the edges, they were submerged in the AOES solution and left to respond for seven hours at room temperature. The solution was initially transparent and colorless, but it progressively turned blue before returning to its original state. Following the reaction, the copper wires’ edges became black. They were carefully taken out of the AOES, cleaned with DI water, and put in a beaker. The beaker was covered with plastic wrap, punctured to let out the water vapor, and dried for 12 h at 70 °C in an oven. After seven hours of etching, the resultant copper wires, known as Cu-CuO fibers, were used in later reactions.

### 2.3. Preparation of Cu-CuO/PEDOT Electrode

A solution was made by mixing 200 µL of EDOT with 20 mL of DI water, which contains 0.5 M H_2_SO_4_ and 0.2 g of sodium dodecyl sulfate (SDS) to facilitate the vapor-phase polymerization of PEDOT on the Cu-CuO edges of wires. The polymerization procedure was then carried out using this solution. Under carefully monitored circumstances, the Cu-CuO wires were exposed to the prepared solution’s vapor, which caused chemical vapor deposition of PEDOT polymer on the wires’ surface. For later sensing applications, the vapor-phase polymerization technique made it easier for PEDOT to cover the Cu-CuO wires uniformly, obtaining improved electrical conductivity and guaranteeing effective signal transduction. The time of polymerization is important for the flexibility, electrical conductivity, and thickness of the film.

### 2.4. Synthesis of CuMPES

The gel electrolyte (PVA/LiCl) was used at the edges of the Cu-CuO/PEDOT wires to create CuMPES, then they were encapsulated in a microneedle for gas detection. Polyvinyl alcohol (PVA) was dissolved in DI water, and then the lithium chloride (LiCl) was added at 80 °C until a translucent solution was obtained. This process created the PVA/LiCl electrolyte solution. We applied this electrolyte solution uniformly to the edges of the Cu-CuO/PEDOT wires after the electrolyte cooled down to room temperature, followed by drying. Subsequently, a controlled environment was created by precisely positioning and closing the coated wires inside the microneedle. To optimize parameters like electrolyte content and microneedle size for increased sensitivity and selectivity, other conductive ends of the wires were linked to a measurement setup for testing and calibration. Applications such as different gas sensing were investigated, utilizing electrochemical performance to confirm real-world testing situations relevant to environmental monitoring. Through this synthesis technique, accurate and dependable gas microsensor for non-invasive environmental monitoring is produced.

## 3. Characterization Results

### Morphological Characterization

**Figure 1:**
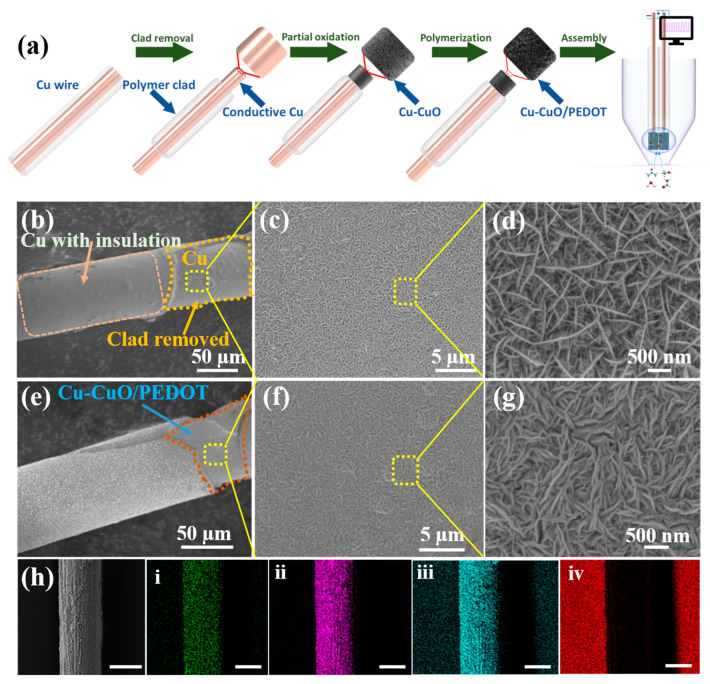
(**a**) Schematic of synthesis and working mechanism of CuMPES. (**b**) SEM image of the Cu wire with cladding removed from the edge. (**c**,**d**) The low- and high-resolution SEM images of CuO on the pristine Cu wire’s edge after exposure to an oxidizing agent. (**e**) SEM image of Cu wire with Cu-CuO/PEDOT edge. (**f**,**g**) The low- and high-resolution SEM images of Cu-CuO/PEDOT at the active edge. (**h**) SEM and corresponding elemental distribution energy spectra of (**i**–**iv**) S, Cu, O, and C, respectively (scale bar: ~50 µm).

Figure 1a shows the synthesis procedure and working mechanism of the CuMPES. Briefly, the polymer cladding from the edges of copper wires was removed using MEK stripping solution at a suitable concentration. Following this, the exposed edges were oxidized to form Cu-CuO layers, obtaining an enhanced sensitivity to the target analytes. Subsequently, the polymerization of PEDOT on the Cu-CuO surfaces was conducted via vapor-phase polymerization, optimizing electrical conductivity, and facilitating signal transduction. Afterward, the edges were coated with a PVA/LiCl gel electrolyte, and the prepared capacitive sensor was encapsulated in a microneedle, ensuring precise positioning and stability during gas sensing. At each step of the preparation, the prepared electrodes were characterized using scanning electron microscopy (SEM), X-ray diffraction (XRD), X-ray photon spectroscopy (XPS), and Raman spectroscopy.

Figure 1b–d show the SEM images of the sample obtained by polymer etching from the edge and the surface oxidation of copper wire using an alkaline etching solution. Figure 1b displays the removal of polymer cladding from one end of the Cu microwires where the rest of the part was not exposed to the etching agent, to keep it as an insulator that can prevent short circuits while encapsulating the electrodes in the microneedle.

Figure 1c,d display the low- and high-resolution SEM images of CuO, respectively. After the PEDOT polymerization on the CuO, the corresponding SEM image revealed a notable change in the sharpness of the edges. Before polymerization, the edges of the CuO exhibited sharp and well-defined features. However, following the polymerization process, these sharp edges became visibly blunted or rounded in appearance (Figure 1f,g).

This change in edge morphology indicates that the PEDOT polymer’s deposition andcoating on the surface of the CuO nanowalls, showing a smoother surface profile. The blunting of the edges suggests the formation of a conformal polymer coating, which may have filled in any gaps or irregularities present on the surface. This observation highlights the transformative effect of the polymerization process on the CuO nanowalls’ surface morphology and structure, demonstrating the effective coating of PEDOT and the integration of the polymer onto the nanostructured substrate. To further confirm the uniform deposition of PEDOT on the Cu-CuO surface, EDS mapping was conducted. Figure 1h reveals a uniform distribution of carbon (C), sulfur (S), copper (Cu), and oxygen (O) across the surface of the Cu-CuO/PEDOT microelectrode. This homogeneous distribution suggests the successful integration of PEDOT onto the CuO nanostructure, resulting in a composite with well-dispersed elements.

**Figure 2:**
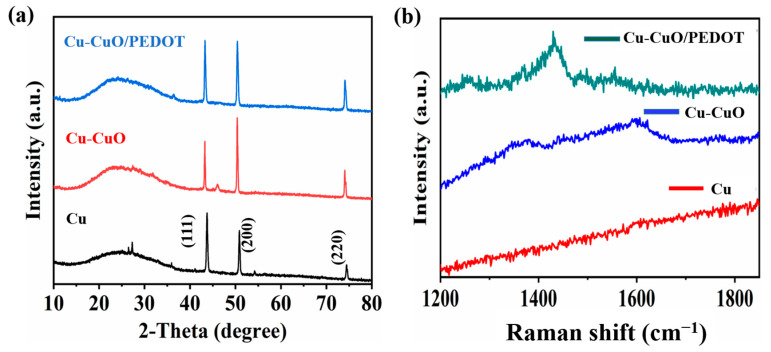
XRD patterns (**a**) and Raman spectra (**b**) of Cu, Cu-CuO, and Cu-CuO/PEDOT electrodes.

The XRD analysis performed on as-prepared Cu wires revealed distinct diffraction peaks at room temperature, as depicted in Figure 2a. These peaks, observed at 43.4°, 50.5°, and 74.2°, correspond to the (111), (200), and (220) crystal planes, respectively, of a face-centered cubic (FCC) lattice structure of pure crystalline copper [33]. Upon the formation of Cu-CuO, there was a notable increase in the intensity of the (220) peak compared with that of pristine Cu. This increase in intensity suggests the presence of the CuO phase alongside the metallic copper. The enhancement in the (220) peak intensity implies a preferential orientation or higher crystallinity of the CuO phase within the composite. The disappearance of peaks between 25° and 30° in the XRD pattern after oxidizing the Cu wire suggests the transformation of the material into CuO. Additionally, the appearance of a peak between 40° and 45° in the XRD pattern of Cu-CuO indicates the presence of a crystalline CuO phase. This shift in peak position reflects the change in crystal structure and phase composition induced by oxidation [34]. Subsequently, when incorporating PEDOT on the Cu-CuO to form the Cu-CuO/PEDOT composite, the XRD analysis revealed the persistence of diffraction peaks characteristic of Cu-CuO, indicating that the overall structure and phase composition of the Cu-CuO remain almost unchanged.

The Raman spectra of the pure Cu wire, Cu-CuO, and Cu-CuO/PEDOT electrodes reveal distinct structural and compositional properties. The pure Cu wire lacks peaks in the range of 1200–1800 cm^−1^, indicating the absence of notable Raman-active vibrational modes as described in Figure 2b. The Cu-CuO electrodes exhibit low-intensity peaks at 1370.94 cm^−1^, 1441.84 cm^−1^, 1563.3 cm^−1^, and 1600.31 cm^−1^, attributed to the formation of CuO on the surface [35,36]. Additional peaks at 1249.51 cm^−1^, 1360 cm^−1^, 1429.6 cm^−1^, 1485.5 cm^−1^, and 1555.7 cm^−1^ exist in the Raman spectrum of Cu-CuO/PEDOT electrode, corresponding to the vibrational modes of the PEDOT polymer, including C-C stretching and various bonds within the polymer chain confirming the uniform coating of the polymer thin layer [37,38].

**Figure 3:**
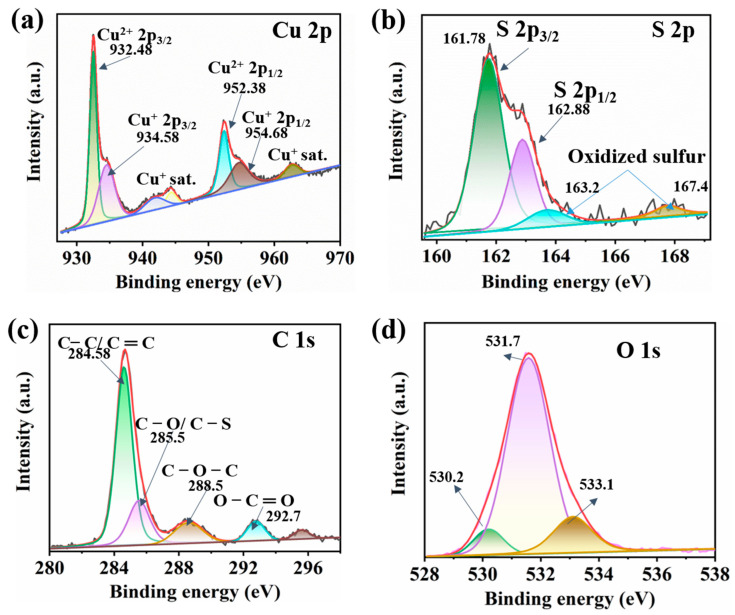
Deconvoluted XPS spectra showing the (**a**) Cu^2+^ 2p_3/2_, Cu^2+^ 2p_1/2_; (**b**) S 2p_3/2_, S 2p_1/2_; (**c**) carbon peaks; and (**d**) oxygen peaks in Cu-CuO/PEDOT electrode.

To further elucidate the chemical composition and surface state of the Cu-CuO/PEDOT electrode, XPS analysis was conducted, allowing for the detailed characterization of the elemental components and chemical states of the electrode. Figure 3 presents the structure and chemical state of the Cu-CuO/PEDOT wires’ electrode by XPS. The main elements detected in the sample are carbon (C), oxygen (O), copper (Cu), and sulfur (S). To deeply explore the chemical composition and state of the Cu-CuO/PEDOT electrode, XPS analysis was conducted, enabling a thorough understanding of the elemental components and chemical states within the sample. Figure 3 illustrates the structure and chemical state of the Cu-CuO/PEDOT wires’ electrode analyzed via XPS. The primary elements detected include C, O, Cu, and S. In Figure 3a, the presence of a doublet in the Cu 2p_3/2_ peaks at 932.48 eV and 934.58 eV indicates the coexistence of two oxidation states of copper, Cu^2+^ and Cu^+^, respectively. These distinct chemical states can be attributed to the interaction between copper and the PEDOT coating. The Cu^+^ species, observed at a higher binding energy (934.58 eV), may result from the surface oxidation of copper facilitated by oxygen-containing functional groups in the PEDOT coating. Conversely, the Cu^2+^ species, observed at a lower binding energy (932.9 eV), may arise from charge transfer processes between PEDOT and copper, where PEDOT acts as an electron donor. Additionally, the presence of satellite peaks at higher binding energies (940.2 eV, 944.3 eV, and 964.5 eV) in the Cu 2p_3/2_ spectra suggests additional electronic transitions associated with the chemical environment of copper within the composite, further indicating the influence of the PEDOT coating on the oxidation state of copper [39,40].

Figure 3b shows the deconvoluted S 2p spectrum, consisting of a spin-split doublet, S (p_1/2_, _3/2_), with a well-known energy difference of 1.18 eV, intrinsic line width, and relative intensity (1:2). At least three chemically distinct sulfur species are existed, with lower peaks at 161.7 eV, 162.8 eV, and 163.2 eV, matching well with the spin-split components of the sulfur atoms in PEDOT [41,42]. In Figure 3c, the three peaks of C 1s from PEDOT are located at 284.58, 285.5, and 288.5 eV, attributed to C–C, C–O, and C–O–C, respectively [43,44]. Figure 3d exhibits three peaks at binding energies of 530.2 eV, 531.7 eV, and 533.1 eV in the O 1s region, which could be attributed to adsorbed O and lattice oxygen [45].

## 4. Electrochemical and Sensing Performance of the CuMPES

The CuMPES was encapsulated within a microneedle with a tip diameter of 100 µm, guiding the gas flow towards the microsensor’s surface. Gas molecules interact with the microsensor’s surface upon exposure, leading to measurable changes in electrical performance. The needle design has enhanced sensitivity and selectivity by directing gas to the microsensor. Encapsulation provides mechanical support and stability during gas sensing, ensuring precise positioning and reliable measurement. For the VOC vapors, ethanol, acetone, and ammonia solutions were carefully prepared at different volume percentages (25%, 50%, 75%, and 100%) by diluting the pure chemicals with DI water. Specifically, a 25% solution was attained by mixing 25 mL of the pure chemical with DI water to reach a total volume of 100 mL. The similar procedures were performed for other concentrations. These solutions were subsequently introduced into a controlled chamber to generate vapor-phase concentrations corresponding to the initial volume percentage of the solution. The sensor was then exposed to vapors, with gas concentrations in the chamber assumed to reflect the volume percentages of the prepared solutions.

**Figure 4:**
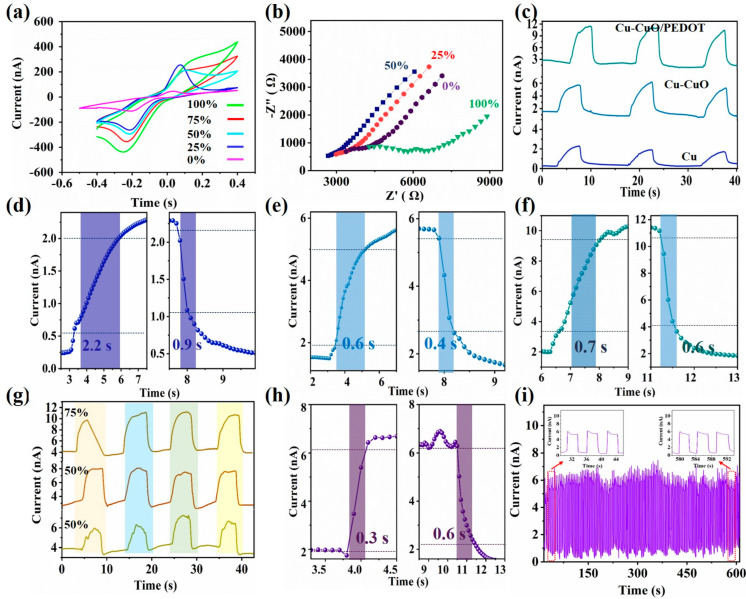
(**a**) Cyclic voltammograms of CuMPES in an inert environment and the presence of ethanol gas at concentrations of 25%, 50%, 75%, and 100% under a scan rate of 5 mV/s. (**b**) EIS profiles of CuMPES in an inert environment and the presence of ethanol gas at concentrations of 25%, 50%, and 100%. (**c**) Current-time (I-T) curves of pristine Cu, Cu-CuO, and CuMPES sensors at zero volts in ethanol gas. Response recovery time of (**d**) pristine Cu, (**e**) Cu-CuO, and (**f**) Cu-CuO/PEDOT sensors in ethanol at a concentration of 50%. (**g**) I-T curves obtained from CuMPES in the presence of ethanol at zero volts with different gas concentrations (25%, 50%, and 75%). (**h**) Response and recovery time of CuMPES in ethanol with a concentration of 25%. (**i**) Stability of CuMPES in ethanol at a concentration of 25% concentration.

We conducted cyclic voltammetry (CV) experiments on CuMPES to evaluate their performance in detecting ethanol at varying concentrations. The CV experiments were performed in an inert environment and in the presence of ethanol at concentrations of 25%, 50%, 75%, and 100%. During the CV experiments, the potential was swept from −0.4 V to 0.4 V at a scan rate of 5 mV/s. We observed a notable increase in the output current of the sensors as the concentration of ethanol gas increased (Figure 4a).

To further confirm the CV results of the response of the microsensor to varying concentrations of ethanol gas, we performed electrochemical impedance spectroscopy (EIS) experiments at ethanol concentrations of 25%, 50%, and 100% (Figure 4b). The experimental results from electrochemical impedance spectroscopy (EIS) align closely with the logical expectations derived from the observed trends in cyclic voltammetry (CV) experiments. At lower ethanol concentrations of 25% and 50%, where the CV output current increased, impedance decreased as anticipated, indicating improved electrical conductivity of the sensing material. Conversely, at higher ethanol concentrations of 100%, where the CV output current continued to increase, the impedance increased as expected, likely due to saturation effects. These results confirmed the microsensor’s sensitivity to ethanol gas at different concentrations and underscore its potential for accurate and reliable gas detection.

For comparison, three types of microsensors, pristine Cu, Cu-CuO, and Cu-CuO/PEDOT microsensors, were fabricated to investigate their gas sensing performance. To assess their response in gas detection, we conducted chronoamperometric tests, measuring the output current variation as the gas inlet was introduced and subsequently stopped (Figure 4c). A distinct difference in performance of the microsensor is observed. The pristine Cu microsensor exhibited a significantly low output current in a gas environment, indicating a limited sensitivity to the target gas. In contrast, the Cu-CuO microsensor displayed a slightly higher output current compared with the pristine Cu microsensor, suggesting an improved sensitivity attributed to the presence of the CuO layer. However, the most notable improvement was observed with the Cu-CuO/PEDOT microsensor, which demonstrated a significantly higher current output while maintaining an almost similar initial current level without gas. This enhancement in the current output indicates the synergistic effect of the PEDOT coating, which obtains an enhanced electrical conductivity and facilitates efficient signal transduction. The response/recovery time of Cu, CuO, and Cu-CuO/PEDOT in 75% ethanol is found to be 2.2/0.9, 0.6/0.4, and 0.7/0.6 s respectively. Compared with the Cu microsensor, the CuMPES exhibited a significantly faster response (0.9 s vs. 2.2 s), enhancing its ability for rapid detection. While the CuMPES’s response time is a little longer than that of the CuO sensor (0.7 s vs. 0.6 s), it still presents a rapid detection ability. The recovery time of the CuMPES (0.7 s) is competitive, presenting efficient reset capabilities like that of the CuO sensor (0.4 s). However, the CuMPES showed a higher response current compared with both the Cu and CuO sensors, indicating its improved sensitivity and signal strength. The sensitivity of CuMPES is 15.6%, higher than those of the CuO (10%) and Cu (9.3%) microsensors. 

To further confirm the sensitivity and selectivity of the microsensor, chronoamperometric tests were meticulously conducted on CuMPES at a fixed potential of zero volts while exposed to varying concentrations of ethanol gas (25%, 50%, and 75%). The experimental protocol involved systematically introducing and halting the flow of ethanol gas to observe the dynamic response of the microsensor. Upon the initiation of the ethanol gas flow, a sudden increase in output current was observed, indicating the onset of gas detection and the initiation of electrochemical processes at the sensor’s surface. This increase in current was proportional to the concentration of ethanol gas, demonstrating the sensor’s sensitivity to changes in the gas concentration. Subsequently, upon the cessation of gas flow, the output current gradually decreased as the sensor recovered from the gas exposure, eventually returning to baseline levels (Figure 4g). The response/recovery time (0.3/0.6 s), defined as the duration required for the output current to revert to its initial level after gas exposure, was found to be relatively short (Figure 4h). This rapid response/recovery suggests efficient gas adsorption and electron transfer processes at the sensor surface upon exposure to ethanol. The sensor’s ability to quickly detect changes in the ethanol concentration underscores its suitability for real-time monitoring applications where fast response is crucial. This observation underscores the efficiency of the CuMPES in detecting ethanol gas and highlights their potential for real-time monitoring applications where a rapid response and recovery are crucial. To further validate the reliability and repeatability of CuMPES, a cyclic stability test was performed over a period of around 600 s, covering about 120 cycles of exposure to ethanol at a concentration of 50%. The CuMPES delivered a stable performance throughout the testing period, as shown in Figure 4i. The inset of Figure 4i shows the sensor’s response during the initial three cycles and the last three cycles. This comparison demonstrates that the CuMPES’s response remained consistent, indicating its excellent reliability and repeatability over multiple cycles of gas exposure and recovery. The consistent performance highlights the sensor’s robustness and suitability for long-term gas detection applications.

**Figure 5:**
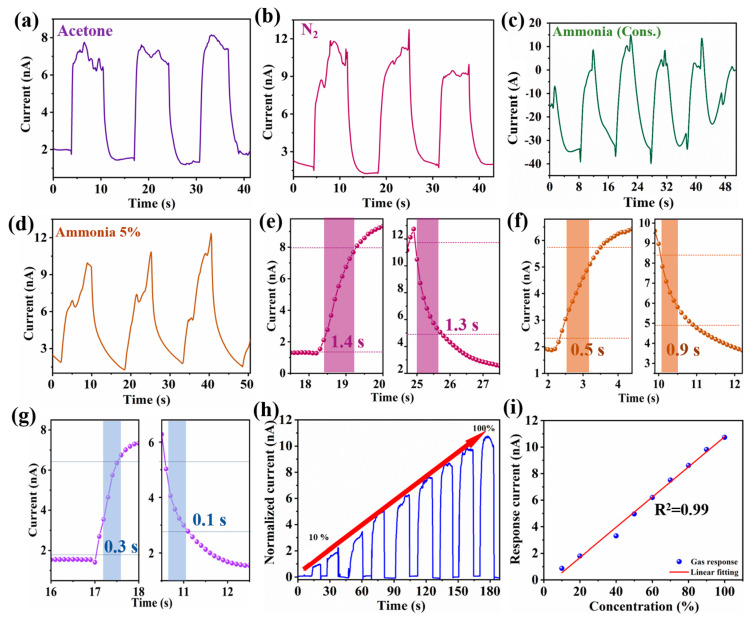
Chronoamperometric test results of CuMPES at zero volts with acetone gas (**a**) and N_2_ gas (**b**) at 50% concentrations. Chronoamperometric test results of CuMPES at zero volts with (**c**) concentrated ammonia and (**d**) 5% ammonia. (**e**–**g**) The response/recovery time of CuMPES in N_2_, NH_3_, and acetone environments, respectively. (**h**) The combined current profile of a CuMPES as a function of the ethanol concentration. (**i**) The linearly fitted relationship between response current and ethanol concentration.

To further confirm the versatility of the sensor, chronoamperometric tests were conducted on CuMPES to assess their performance in sensing acetone and nitrogen (N_2_) gases at a concentration of 50%. The tests involved the controlled introduction and cessation of gas flow while monitoring the resulting current response of the sensor. For nitrogen gas sensing, upon introducing the gas, a notable increase in output current was observed, reaching a maximum value of approximately 12 nA. This surge in current reflects the sensor’s sensitivity to nitrogen gas, with the presence of the gas leading to enhanced electron transfer processes at the sensor interface. Conversely, when the nitrogen gas flow was halted, the output current gradually decreased, eventually returning to baseline levels. The observed current without gas was approximately 2 nA, indicating the baseline level of the current in the absence of gas exposure. Similarly, for acetone gas sensing, an output current of approximately 8 nA was achieved upon gas introduction, highlighting the sensor’s ability to detect acetone gas at the specified concentration (Figure 5a,b). These results demonstrate the responsiveness and selectivity of the CuMPES to acetone and nitrogen gases, with distinct current responses observed for each gas. Afterward, the CuMPES was exposed to concentrated ammonia gas, and unexpected current profiles were detected. A complicated interaction between the ammonia gas and the sensor’s surface is suggested by the high-intensity reversal of the current direction towards the negative direction, reaching −40 nA when exposed to concentrated ammonia gas (Figure 5c). This large negative current may result from ammonia molecules being reduced at the sensor surface, which would produce electrons and a net current flow in the opposite direction. The observed decrease in current magnitude upon stopping the gas flow, while still indicating a negative direction and reaching roughly 10 nA, suggests that the ammonia gas has remained on the sensor surface, causing electron transfer activities to continue, albeit at a slower pace. Conversely, upon exposure of the CuMPES to ammonia gas at a concentration of 5%, a positive current response measured around 10 to 12 nA was noted (Figure 5d). The observed positive current implies an alternative mechanism of interaction between the sensor’s surface and the reduced ammonia gas concentration. Due to the improved electron transfer mechanisms, the ammonia molecules may experience adsorption and desorption processes on the sensor’s surface at this reduced concentration, changing the surface charge and producing a positive current response. The obtained results provide quantitative information about the gas concentrations and underscore the potential of CuMPES for diverse gas sensing applications in various environments. The response/recovery time of the CuMPES for different gases, including ammonia, acetone, and N_2_ are found to be 0.5/0.9, 0.3/0.1, and 1.4/1.3 s, respectively, as displayed in Figure 5e–g.

The combined current profile of a CuMPES as a function of ethanol concentration from 10% to 100% is shown in Figure 5h. The y-axis shows the matching output currents of the sensor in nano amperes (nA), and the x-axis shows the time with ethanol concentration in percentage, ranging from 10% to 100%. There is a noticeable pattern of rising output currents with increasing ethanol concentration, from about 1 nA at 10% ethanol concentration to around 10 nA at 100% ethanol concentration. This suggests that the sensor responds to ethanol gas in a dose-dependent manner, with higher concentrations producing larger output currents. A similar outcome can be obtained using N_2_, ammonia, methanol, acetone, and N_2_.

The fitted relationship between the gas response and ethanol concentration is shown in Figure 5i. The fitted gas response values from the regression analysis are shown on the y-axis, while the x-axis shows the ethanol concentration in percentage, with a range of 10% to 100%. A coefficient of determination (*R*^2^) value of 0.99 from the regression analysis showed that the model suited the experimental data quite well. The trend in the experimental data is captured by the fitted curve, which indicates a constant rise in the gas response as the ethanol concentration rises. The sensor’s sensitivity to ethanol gas at various concentrations is quantitatively described by this fitted relationship, which makes it possible to predict the microsensor’s response with accuracy at any given ethanol concentration within the tested range. The comparison of proposed microsensor with recently reported sensors is shown in Table 1, indicating that the response/recovery time of proposed microsensor is shorter than those of the recently reported sensors.

## 5. Conclusions

In conclusion, the CuMPES was fabricated by the chemical oxidation of pristine Cu microwire followed by the vapor-phase polymerization of the conducting polymer, signifying the development in gas sensing technology, contributing to improved sensitivity, selectivity, and reliability for environmental monitoring applications. Through comprehensive characterizations, the structural and compositional characteristics of the CuMPES were explained, emphasizing its unique Cu-CuO/PEDOT nanocomposite composition. The incorporation of PEDOT onto the Cu-CuO surface considerably improved the microsensor’s performance, as demonstrated by the perceived threefold increase in the response current compared with pure Cu sensors. The ultra-fast response and recovery of 0.3/0.6 s, combined with the high sensitivity, highlight the microsensor’s significant abilities for detecting various target gases at low concentrations. Additionally, the encapsulation of the microsensor within a microneedle offers a crucial directional flow for competent gas detection, improving sensitivity and selectivity by confirming that only gases from the abrupt environment directly intermingle with the sensor surface. This design feature not only improves the sensor’s performance but also boosts its mechanical stability and reliability in gas sensing operations.

## Figures and Tables

**Table 1:** Comparison of the proposed microsensor with recently reported sensors.

Sensor Type	Material	Target Gas	Method	Operating Temperature (°C)	Response Time (s)	Recovery Time (s)
Resistive [46]	ZnO: Mg (NW)	C_2_H_5_OH	Hydrothermal, CVD	300	15	20
Resistive [47]	SnO_2_ (NW)	C_2_H_5_OH	VLS, CVD	300	15	20
Resistive [48]	WO_2_ (NW)	NH_3_	CVD	25	40	50
Resistive [49]	SnO_2_ (SNW)	NH_3_	VLS, Hydrothermal, CVD	300	20	25
FET [50]	In_2_O_3_/PANI (NPs)	NH_3_	Electrospinning, Chemical Precipitaion	25	500	500
Chemiresistive [51]	MoO_3_ (NPs)	NH_3_	Chemical Precipitation, Hydrothermal	400	60	180
Capacitive [52]	Cu-doped ZnO	C_2_H_5_OH, NH_3_	SGM, SSR	25	13	33
Resistive [53]	NiO@LaFeO_3_	C_2_H_5_O	CP, SSR	240	2	9
Capacitive (This Work)	Cu-based	C_2_H_5_OH	-	25	2.2	0.9
Capacitive (This Work)	Cu-CuO	C_2_H_5_OH	PO	25	0.6	0.4
Capacitive (This Work)	Cu-CuO/PEDOT	C_2_H_5_OH, CH_3_OH, C_3_H_6_O, NH_3_, N_2_	PO, VPP	25	0.7	0.6

## Data Availability

The data are contained within the article.
